# Experimental and In-Silico Computational Modeling of Cerium Oxide Nanoparticles Functionalized by Gelatin as an Eco-Friendly Anti-Corrosion Barrier on X60 Steel Alloys in Acidic Environments

**DOI:** 10.3390/polym14132544

**Published:** 2022-06-22

**Authors:** Hany M. Abd El-Lateef, Mohamed Gouda, Mai M. Khalaf, Manal A. A. Al-Shuaibi, Ibrahim M. A. Mohamed, Kamal Shalabi, Reda M. El-Shishtawy

**Affiliations:** 1 Department of Chemistry, College of Science, King Faisal University, Al-Ahsa 31982, Saudi Arabia; mmkali@kfu.edu.sa (M.M.K.); 218038147@kfu.edu.sa (M.A.A.A.-S.); 2 Chemistry Department, Faculty of Science, Sohag University, Sohag 82524, Egypt; imaashour20080@yahoo.com; 3 Department of Chemistry, College of Science and Humanities in Al-Kharj, Prince Sattam Bin Abdulaziz University, Al-Kharj 11942, Saudi Arabia; dr-kamal@mans.edu.eg; 4 Chemistry Department, Faculty of Science, Mansoura University, Mansoura 11432, Egypt; 5 Chemistry Department, Faculty of Science, King Abdulaziz University, Jeddah 21413, Saudi Arabia; relshishtawy@kau.edu.sa; 6 Dyeing, Printing and Textile Auxiliaries Department, Institute of Textile Research and Technology, National Research Centre, 33 EL Buhouth St., Dokki, Giza 12622, Egypt

**Keywords:** ceria, coated films, corrosion protection, X60 steel alloys, gelatin, theoretical study

## Abstract

An eco-friendly and a facile route successfully prepared novel cerium oxide nanoparticles functionalized by gelatin. The introduced CeO_2_@gelatin was investigated in terms of FE-SEM, EDX, TEM, chemical mapping, FT-IR, and (TGA) thermal analyses. These characterization tools indicate the successful synthesis of a material having CeO_2_ and gelatin as a composite material. The prepared composite CeO_2_@gelatin was used as an environment-friendly coated film or X60 steel alloys in acidizing oil well medium. Moreover, the effect of CeO_2_ percent on film composition was investigated. LPR corrosion rate, *E_ocp_*-time, EIS, and PDP tools determined the corrosion protection capacity. The CeO_2_@gelatin composite exhibited high protection capacity compared to pure gelatin; in particular, 5.0% CeO_2_@gelatin coating film shows the highest protection capacity (98.2%), with long-term anti-corrosive features. The % CeO_2_@gelatin-coated films formed the protective adsorbed layer on the steel interface by developing a strong bond among nitrogen atoms in the CeO_2_@gelatin film and the electrode interface. Surface morphology using FESEM measurements confirmed the high efficiency of the fabricated CeO_2_@gelatin composite on the protection X60 steel alloys. DFT calculations and MC simulations were explored to study the relations between the protection action and the molecular construction of the coated systems, which were in good alignment with the empirical findings.

## 1. Introduction

X60 steel alloys are reported to be used in numerous industries, including household applications, the auto industry, equipment assembly, and chemical manufacturing, owing to their outstanding characteristics and low price [1]. Nevertheless, under acidic circumstances, steel corrosion is a major issue [2]. This is a particular problem in the oil industry, where X60 steel alloys (steel pipelines) are utilized for the pickling process with inorganic acids (for example HCl and other inorganic acids [3,4,5]. Consequently, inspections of the metal corrosion in hydrochloric acid are of manufacturing significance [6].

One of the cheapest and easiest techniques to impede corrosion is to use barrier coatings like organic coating (plastic, paint) or powder. In the literature, many categories of coatings have been described to avoid steel constituents corrosion; coatings based on polymeric materials have been the most extensively utilized owing to their excellent barrier characteristics [7]. The corrosion protection of polymeric coatings results from the coating blocks’ influence and the protection of the corroding locations [8]. The first needs the application of impermeable and thick coatings that avoid the interaction of the metal surface with the aggressive medium (15% HCl solution) [9], while the second includes the distribution of anti-deterioration pigments that hinder corrosion when the defensive barrier influence vanishes [10]. Nevertheless, this method is not easy, where the immediate interaction of some corrosion additives with the defensive conditions might cause undesirable reactions that negatively influence the coating block characteristics. Therefore, the corrosion additives could be loaded into micro/nano-carriers with respectable compatibility with the coating, predominantly inorganic ones that might also assist as plasters, improving the barrier coating features. To better inspect this route, it is significant to use transporters that intelligence particular incentives and that release the inhibitor when required, leading to a keener corrosion protection influence, thereby providing consistent long-term corrosion inhibition [11].

Recently, chromate-comprising pigments have been utilized as corrosion additives because of their good compatibility and high efficacy with coatings; in addition, these pigments have been shown to be the most appropriate to impart the self-healing capability in coatings. Nevertheless, the poisonous nature of pigment-based chromate materials has limited their use, and there exists currently a serious effort to discover substitutions that are more eco-friendly [12,13]. In this regard, the attention to biodegradable and renewable corrosion additives has been elevated significantly [14,15].

CeO_2_ (Cerium oxide, ceria) has received noteworthy attention as a stabilizer for polymeric coating systems because of some significant properties, for example diverse morphologies, chemical steadiness, and reduced poisonousness and structural characteristics [16,17]. Although very steady, predominantly in an alkaline medium, cerium oxide is still capable of releasing Ce ions in acidic systems like the ones produced when steel corrosion occurs [18]. These Ce ions could then interact with OH anions developing protecting hydroxide films that are extensively described as corrosion inhibitors of the cathodic type. Consequently, cerium oxide nano-particles may correspondingly have a significant role in corrosion protection [19]. Furthermore, the distribution of ceria NPs in different paints has led to improved corrosion inhibition owing to the construction of an ultrafine system [20] with declined porosity, and ceria may as well assist as a transporter for the filling of corrosion additives because of its chemical (and at times porous) nature [21].

Inhibitors have developed as a green possibility and are commonly reflected for the protection of diverse alloys and metals [22,23]. Among these, gelatin, which has been utilized in biological systems owing to its renewable origin and good biodegradability, has garnered some consideration [24,25]. Gelatin has been described as an eco-friendly inhibitor for steel corrosion in the harsh acidic medium [26,27], but its filling into carriers to develop keener anti-corrosion inhibitors for polymeric coating systems applied for steel corrosion protection of the acidic pickling conditions has certainly not been described to this point. Therefore, this paper aims at investigating novel anti-corrosion films based on ceria NPs functionalized by gelatin prepared by a facile method. The introduced CeO_2_@gelatin was inspected in terms of FE-SEM, EDX, TEM, and chemical mapping, FT-IR, and (TGA) thermal analyses. The ceria NPs functionalized with gelatin were homogenously dispersed into an epoxy construction that was applied to coat the X60 steel substrate. The LPR corrosion rate and electrochemical measurements were used to estimate the protection performance. Furthermore, the surface morphology of the X60 steel was inspected by a scanning electron microscope (SEM). Finally, DFT calculations and MC simulations were presented to demonstrate the protection mechanism.

## 2. Experimental Part

### 2.1. Preparation of Cerium Oxide Nanoparticles Loaded onto Gelatin (CeO_2_@gelatin) Nanocomposite Films

Cerium oxide nanoparticles (CeO_2_NPs) were loaded onto an aqueous gelatin to make CeO_2_@gelatin films. A combined solution of gelatin (1.5 g/100 mL) and CeO_2_NPs was added to 5% of gelatin weight (CeO_2_ NPs:gelatin solution = 1:20) and was stirred in a three-necked flask for 2 h in the absence of light. CeO_2_@gelatin films with a thickness of 0.8 mm were cast in a Teflon Petri dish at 60 °C for 24 h. The prepared film was desalted with deionized water and dried for 72 h at 50 °C, yielding CeO_2_@gelatin nanocomposite films.

### 2.2. Preparation of CeO_2_@gelatin Coating Films

X60 steel alloy samples utilized as substrate supplies were polished gradually with emery papers No. 500-1600 and then dipped in ethanol, acetone, and bidistilled H_2_O for 5 min and then air desiccated. The X60 steel alloy surface coating was organized by using a dip-coating technique. The electrode substrate was dipped in the sol for 2 min and at a withdrawn speed of 10 mm min^−1^. After air dehydrating, the specimen was dried out in the oven at 80 °C for 15 min. Finally, the process from immersion to dehydrating was reiterated twice to increase the thickness of the coatings. The samples were coated with diverse CeO_2_ percentages (1.0, 2.0, 3.0, 4.0, 5.0 and 10.0% of CeO_2_@gelatin coating, respectively.

### 2.3. Corrosion Protection Measurements

All electrochemical experiments were performed in an electrochemical cell containing 3-electrode systems of Pt-sheet, Ag/AgCl/KCl_(sat)_ (silver/silver chloride), and the uncoated and coated X60 steel alloy specimens as a counter electrode, as a reference and working electrode, respectively. EIS experiments were carried out to estimate the corrosion protection of uncoated and coated specimens in 15% HCl solution at 50 °C in 100 kHz to 1.0 Hz as a frequency range. The potentiostat/galvanostat/ZRA (Gamry 600, Warminster, PA 18974 USA) apparatus was applied for EIS investigation. The EIS experiments were accompanied at 10 points/decade (scan frequency rate) with an open circuit potential (*E_OCP_*) for 45 min and the RMS signal was changed between 2.0 and 10.0 mV. PDP experiments were implemented to examine the corrosion protection performance of the uncoated and coated X60 steel alloy specimens. The PDP experiments were achieved in the potential range of ±250 mV vs. *E_OCP_* at a scan rate of 0.2 mV/s. All tests were completed at least twice.

### 2.4. Characterization Techniques

An FTIR spectrophotometer manufactured by BRUKER (Ettlingen, 76275 Germany) was utilized to investigate the FTIR spectroscopy and study the effective functional groups of the prepared materials with and without CeO_2_ in the range 400.0–4000.0 cm^−^^1^. The thermal stability character of the prepared materials was studied by TGA Instruments (New Castle, DE, USA) for both gelatin and CeO_2_@gelatin binary composites, and the range of temperature was started from room temperature to 700 °C in a flow of O_2_ gas. The studied CeO_2_@gelatin composite has oxide and organic contents, and these parts have different thermal properties. Thus, oxygen was used as a flowing gas to clarify this difference. Moreover, field emission scanning electron microscopy (FESEM) using (JOELF, Tokyo, Japan) with a voltage of around 20 kV was applied to identify the morphological, chemical, and structural morphology of CeO_2_@gelatin. Moreover, a Jeol-1230 electron microscope operating at 200 KeV was used to investigate the nano-size and confirm the morphology of the fabricated CeO_2_@gelatin samples.

### 2.5. Surface Characterization

In order to detect the changes in the morphology of the X60 steel alloy surfaces, the corroded surfaces of the uncoated and coated before and after dipping in the aggressive medium (15% HCl solution) for 24 h were categorized by FESEM (FE-SEM-Nova Nano-450, Austin, TX USA).

### 2.6. Computational Details

The energy of the CeO_2_, gelatin and CeO_2_@gelatin molecules had been optimized in aqueous media by employing DFT calculations with a basis set BOP with the ab initio, GGA technique and DNP 4.4 accomplished in the Dmol^3^ module in BIOVIA Materials Studio 2017, Dassault Systèmes, Vélizy-Villacoublay, France [28]. The outcomes achieved from DFT calculations involving the lowest unoccupied molecular orbital (*LUMO*), the highest occupied molecular orbital (*HOMO*), the gap energy (Δ*E*), electronegativity (*χ*), hardness (*η*), global softness (*σ*) and the number of electrons transferred (Δ*N*), ∆*E_back-donation_* and dipole moment (*µ*) were calculated as follows [29]:

(1) $$\chi =\frac{-E_{HOMO}-E_{LUMO}}{2}$$

(2) $$\eta =\frac{1}{\sigma }=\frac{E_{LUMO}-E_{HOMO}}{2}$$

(3) $$\Delta N=\frac{\varphi -\chi_{inh}}{2(\eta_{Fe}-\eta_{inh})}$$

(4) $$\Delta E_{back-donation}=\frac{-\eta }{4}$$

where, *φ* is the function work of Fe (110), χ*_inh_* signifies the inhibitor electronegativity, *η_Fe_* and *η_inh_* are the chemical hardness of Fe (0 eV) and inhibitor, respectively.

In MC simulations, the appropriate adsorption arrangements of the CeO_2_@gelatin molecule on the Fe (110) surface were attained via the adsorption locator module in the Materials Studio V.7.0 software [30]. Initially, the energy of the adsorbate molecules had been optimized by the proceeding COMPASS module [31]. Subsequently, the adsorption of the CeO_2_@gelatin, Cl^−^ ions, hydronium ions, and water molecules with the surface of Fe(110) was implemented in a simulation box (37.24 Å × 37.24 Å × 59.81 Å) [32].

## 3. Results and Discussions

### 3.1. Morphology and Chemistry Analysis

**Figure 1:**
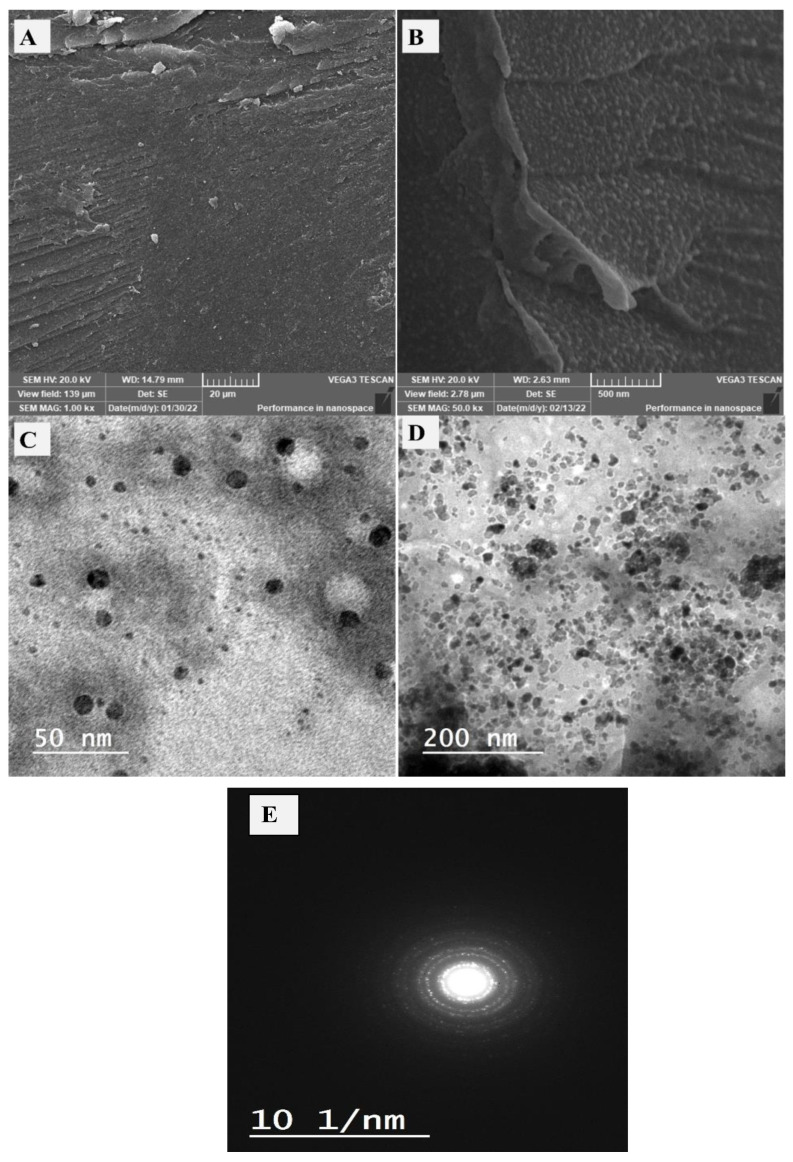
FESEM of gelatin (**A**) and 5% CeO_2_@gelatin (**B**), and TEM images of 5% CeO_2_@gelatin sample at low and high magnification (**C**,**D**, respectively) and the SAED pattern (**E**) of the prepared nanocomposite.

**Figure 2:**
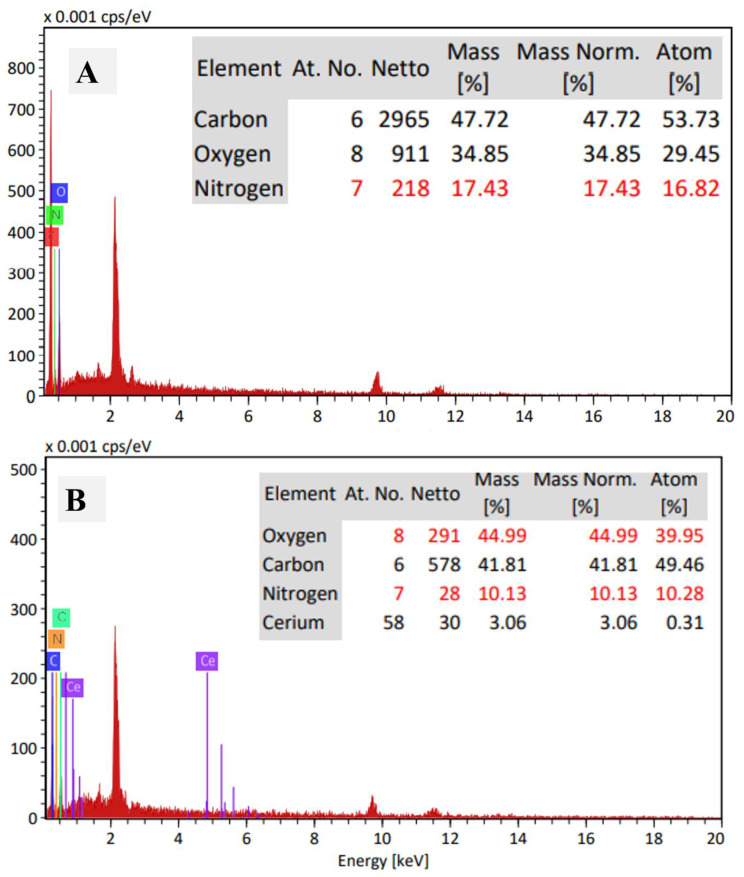
EDX of gelatin (**A**) and 5% CeO_2_@gelatin (**B**), and the inset is the chemical elemental analysis based on EDX peaks analysis.

The morphology of the utilized gelatin and prepared CeO_2_@gelatin was investigated by FESEM as shown in Figure 1A,B, in addition to TEM images of the synthesized CeO_2_@gelatin material (Figure 1C,D). For FESEM images, the gelatin image (Figure 1A) has a smooth and homogeneous surface with no clear boundaries or isolated particles, which indicates the existence of only organic material. After CeO_2_ incorporation, the surface morphology becomes heterogeneous, and different size particles can be seen, indicating the successful chemical design of the heterogeneous gelatin/CeO_2_ composite in terms of morphology. The found particles in the FESEM image of CeO_2_@gelatin were confirmed by TEM images at different magnifications, as shown in Figure 1C,D for low and high magnification, respectively. Nanoscale size particles could be detected in both TEM images. The selected area electron diffraction (SAED) pattern shown in image E indicates a typical poly nanocrystalline nature of CeO_2_ that has a diameter of 10 (1/nm). Therefore, the FESEM and TEM images proved that the prepared material has a heterogeneous character and contains nanoscale-sized particles. To indicate the chemistry of the found particles and discuss the chemical contents of the prepared material, EDX analysis was studied for pure gelatin and CeO_2_@gelatin, as shown in Figure 2A,B, respectively. For gelatin without CeO_2_, the atomic percentages of C, O, and N were found at 53.73, 29.45, and 16.82%, respectively. After CeO_2_ incorporation, the atomic percentages of C, O, Ce and N were found at 49.46, 39.95, 0.31, and 10.28%, respectively. The decrease of C and N contents in addition to the increase in the percentage of O indicates the change of chemical content of the gelatin to theoxide of Ce.

**Figure 3:**
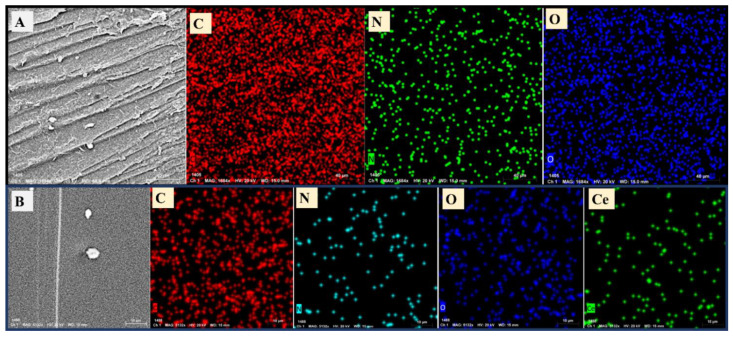
Chemical mapping by FESEM of gelatin (**A**) and 5% CeO_2_@gelatin (**B**), and the mapping images include the mapping of C, N, O for the gelatin sample and C, N, O, and Ce for the 5% CeO_2_@gelatin sample.

The elemental mapping was additionally made for gelatin and CeO_2_@gelatin as shown in Figure 3A, and 3B, respectively. The decrease of O and N contents could be seen and confirmed in chemical mapping as found in EDX-analysis. Additionally, the map of Ce was extended to all the investigated areas like what was found in O but with less quantity. The O-contents was higher than that of Ce because O is expected to be found in gelatin and CeO_2_ in contrast to Ce, which should be present only in CeO_2_ content. The FESEM, TEM, EDX, and chemical mapping indicate the preparation of CeO_2_@gelatin material.

**Figure 4:**
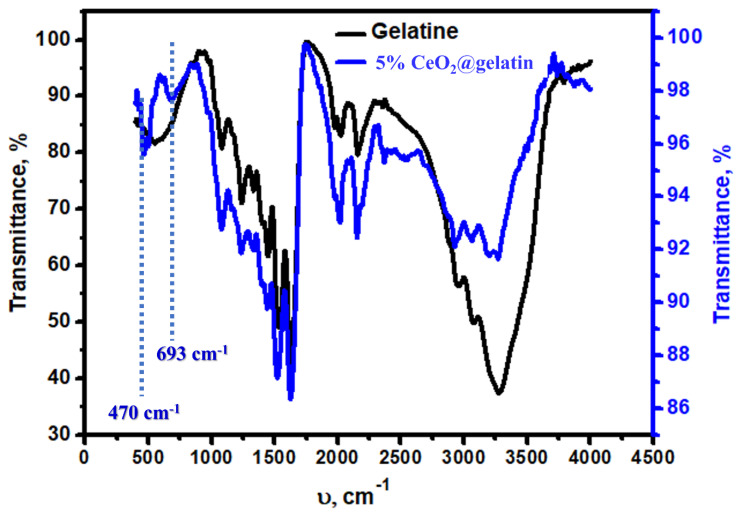
FT–IR analysis of gelatin and 5% CeO_2_@gelatin samples.

The chemistry of bonds in the synthesized sample was studied via FT-IR spectroscopy, as displayed in Figure 4. Both samples (gelatin and CeO_2_@gelatin) have similar characteristic FT–IR peaks. The typical peaks at 3267, 3071, 2950, 2161, and 1526 cm^−1^ of the gelatin sample were found at CeO_2_@gelatin with a small shift which could be due to the interaction between the Ce-O bonds and the gelatin function groups. Two peaks at 693 and 470 cm^−1^ were found in the CeO_2_@gelatin and were not seen in gelatin, which is attributed to the Ce-O stretching vibration. In short, FT-IR analysis indicates the presence of gelatin function groups in addition to Ce-O bonds for the CeO_2_@gelatin and confirms the successful and proper incorporation of Ce-oxide into the gelatin structure.

### 3.2. Thermal Properties

**Figure 5:**
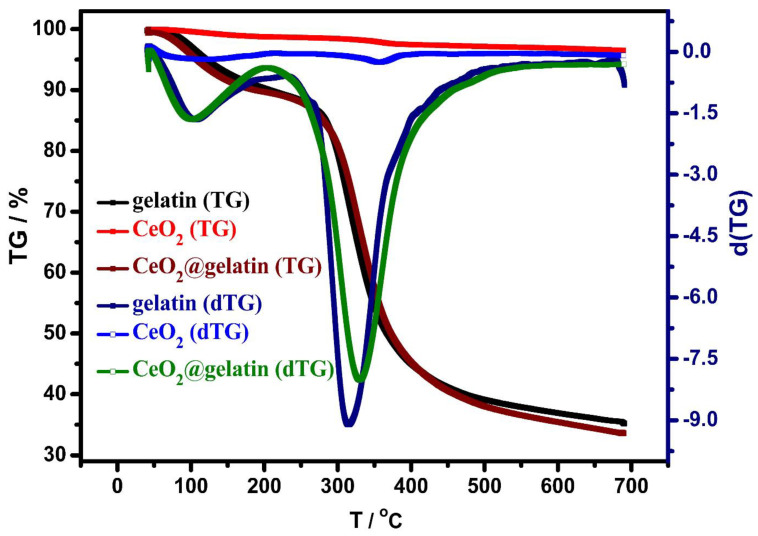
TGA analyses of gelatin, CeO_2_, and 5% CeO_2_@gelatin samples and their dTG analysis.

TGA and DTG analyses were investigated for gelatin, CeO_2_, and CeO_2_@gelatin materials, as shown in Figure 5. The weight loss in the case of CeO_2_ is small at approximately 3.48%, which indicates the thermal stability of CeO_2_ up to the investigated temperature (700 °C). This thermal stability could be due to the difficult transformation or evaporation of the oxide compounds. For gelatin and CeO_2_@gelatin, there are two major exothermic peaks at 102 °C and 317 °C, respectively. These peaks could be due to the humidity removal (adsorption water molecules) and decomposition of organic contents (gelatin). Furthermore, the weight loss was equal to 64.80%, and 66.45% at 700 °C for gelatin and CeO_2_@gelatin, respectively. There is a small shift that could be seen in the second exothermic peak of CeO_2_@gelatin if compared with the gelatin sample, which could be attributed to the interacted organic content with the oxide. This interaction could provide more stability to the organic contents and so a shift to higher temperature was found. The interaction between CeO_2_ and gelatin in CeO_2_@gelatin was in accordance with the shift found in FT-IR peaks as discussed before. The thermal characteristics of CeO_2_@gelatin approve the successful preparation of CeO_2_@gelatin composite.

### 3.3. OCP vs. Time and Tafel Polarization

**Figure 6:**
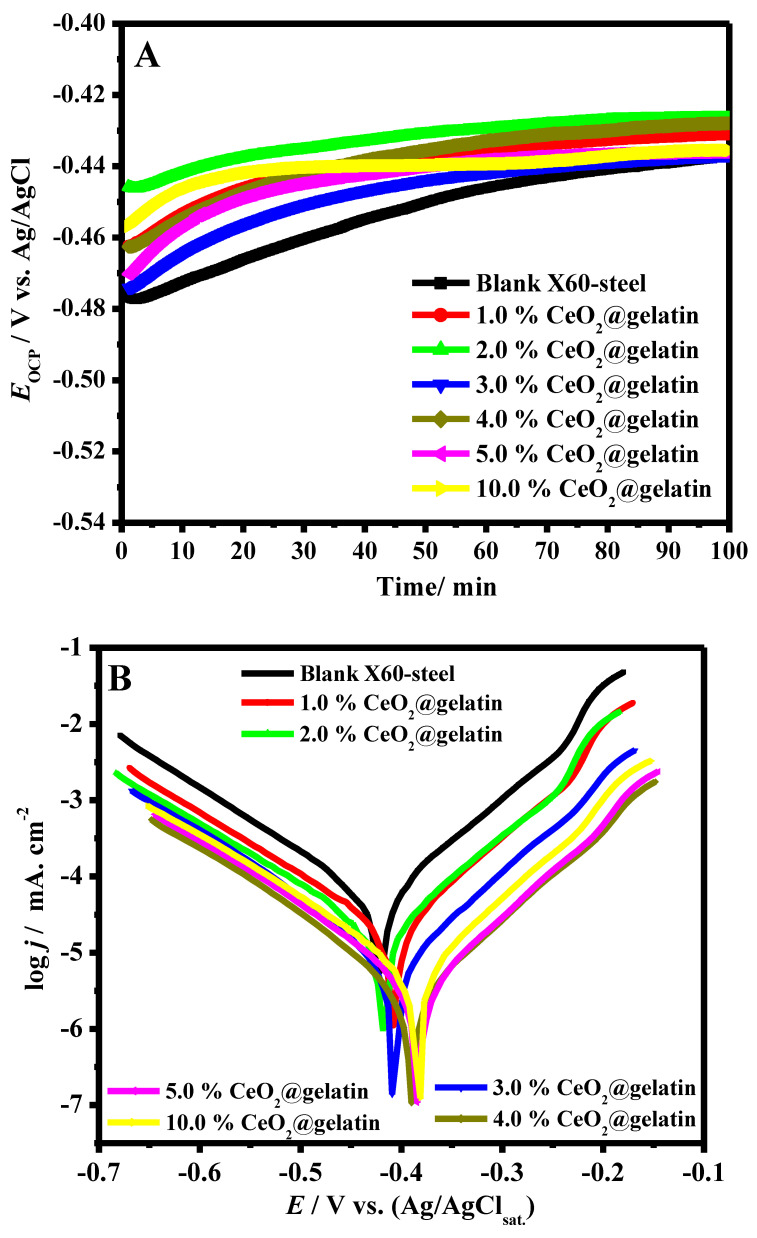
OCP vs. time (**A**) and Tafel plots (**B**) in 15% HCl solution for blank X60–steel and coated samples with different percent of % CeO_2_@gelatin at 50 °C.

It is well recognized that the OCP of a system is a factor utilized as a thermodynamic parameter of electrochemical deterioration in an aggressive solution [33]. In the lack of employed potential, the steel substrate potential might differ with time due to the surface nature of steel, which can be changed because of the anodic and cathodic spontaneous reactions. However, the OCP vs. time could be altered owing to the development of the passive film, oxidation, or resistance [34]. Figure 6A demonstrates the OCP vs. time in 15% HCl solution for blank X60-steel and coated specimens with various ratios of CeO_2_@gelatin at 50 °C. All coated specimens at the initial time of dipping are shifted to more positive potential compared with the pristine X60-steel substrate, which confirms that the samples of coated X60-steel are in the passive state and, consequently, are protected from the chloride corrosive medium [35]. Generally, the early positive potential for CeO_2_@gelatin coated films is associated with the reduction-oxidation process in the gelatin layers. At the start of dipping in the corrosive chloride solutions, OCP values of CeO_2_@gelatin coated films were recorded as being from −0.472 to −0.444 V vs. Ag/AgCl, while the value for blank X60-steel was about −0.476 V vs. Ag/AgCl. This phenomenon indicated that the CeO_2_@gelatin coated films promoted the expansion of a steadier passive layer, possibly in a method similar to anodic protection. This confirms that CeO_2_@gelatin coated films increase the protection of steel corrosion by snowballing the steel nobility.

The PDP diagrams of the blank X60-steel and coated samples with different percentages of % CeO_2_@gelatin were performed after 40 min of soaking in 15% HCl solution at 50 °C, as presented in Figure 6B. By examination of Figure 6B, it is observed that the cathode and anode reactions were affected by the CeO_2_-percent increase in the coatings films, which revealed that these films impede the anodic deterioration of the steel and the cathodic hydrogen evolution [36]. Moreover, the cathodic and anodic branches move towards inferior current densities in the case of coated films, which indicate a decline in the deterioration of the active parts [37].

The parameter values of coated and uncoated specimens, including corrosion current density (*j_cor_*), corrosion potential (*E_cor_*), and the cathodic and anodic Tafel slopes (*β_c_*, *β_a_*) recorded in Table 1 were attained by extrapolating Tafel slopes to *E_cor_*. The protection capacity *(η*/%) values were intended by the measured *j_cor_*, from the uncoated ($j_{cor}^{unc}$) and coated ($j_{cor}^{c}$) surfaces by the following Equation [38]:

(5) $$\mathrm{Protection}\;\mathrm{capacity}/\%=\left(\frac{j_{cor}^{unc}-j_{cor}^{c}}{j_{cor}^{unc}}\right)\times 100$$

The *E_cor_* values from the PDP (Table 1) plots upsurge in the following order: pristine C-steel (−418 mV (Ag/AgCl_(sat)_)) < 3% CeO_2_ coating (−404 mV (Ag/AgCl_(sat)_)) < 4% CeO_2_ coating (−398 mV (Ag/AgCl_(sat)_)) < 5% CeO_2_ coating (−384 mV (Ag/AgCl_(sat)_)) <10% CeO_2_ coating (−378 mV (Ag/AgCl_(sat)_)); this shift in *E_cor_* towards positive direction exposes the upsurge in corrosion resistance. After insertion of CeO_2_ nanoparticles in a gelatin matrix, the substrates display an inferior *j_cor_*, demonstrating a lessening in the corrosion rate of the CeO_2_@gelatin specimens. That is, the CeO_2_ nanoparticles functionalized by gelatin coatings deliver improved corrosion protection for the steel surface in 15% HCl solution. The 5.0% CeO_2_@gelatin coating film shows the lowest *j_cor_* among all the specimens, inferring the greatest performance of this film resulting from its uniform interface construction. The retained protection effects could be attributed to the dense surface construction and enhanced adhesion after CeO_2_ insertion in the polymeric matrix, thus decreasing the corrosive ion penetration. Decreasing the percent of CeO_2_/gelatin will decrease the occurred adhesion because as polymer (gelatin) contents increase, the adhesion is improved. At lower CeO_2_ contents, the interaction between CeO_2_ and gelatin would be small. Therefore, the best content of CeO_2_ was found at 5%. The Tafel slopes (*β_c_* and *β_a_)* values in the case of coated substrates were found to be higher than in the case of pristine C-steel, which supports that the CeO_2_@gelatin coating layers alter the mechanism of the hydrogen evolution reaction [28]. Moreover, *β_a_* are also found to be higher than the corresponding *β_c_*.

The reported data in Table 1 showed a noteworthy decline in *j_cor_* values in the occurrence of CeO_2_ in the gelatin matrix compared to the surface coated with pristine gelatin, and this diminution prevails with cumulative CeO_2_ percent up to 5% CeO_2_ and then increases. The *j_cor_* values decrease in the following order: pristine C-steel (398.1 µA cm^−2^) > pure gelatin-coating (159.2 µA cm^−2^) > 1% CeO_2_ coating (118.23 µA cm^−2^) > 2% CeO_2_ coating (73.25 µA cm^−2^) > 3% CeO_2_ coating (25.47 µA cm^−2^) > 4% CeO_2_ coating (9.95 µA cm^−2^) > 5% CeO_2_ coating (7.16 µA cm^−2^) < 10% CeO_2_ coating (14.33 µA cm^−2^). This proposes that CeO_2_@gelatin diminishes the rate of steel corrosion. The decrease in *j_cor_* values in the existence of CeO_2_ in the gelatin matrix could be ascribed to the hindering of the active places present on the metal interface [39]. The supreme 98.2% corrosion protection capacity was recorded in the case of 5.0% CeO_2_@gelatin coated film. The steel surface coated with CeO_2_@gelatin exhibited respectable corrosion resistance, possibly owing to the worthy dispersion of CeO_2_ in the polymeric matrix. The use of gelatin functionalized CeO_2_ improves the barrier characteristics owing to superior crosslinking of the CeO_2_@gelatin composite. Eventually, the occurrence of ceria nanoparticles with gelatin decreases the pores obtainable for H_2_O uptake and removes conductive routes in the film coating, improvising its barrier features.

### 3.4. EIS Studies

**Figure 7:**
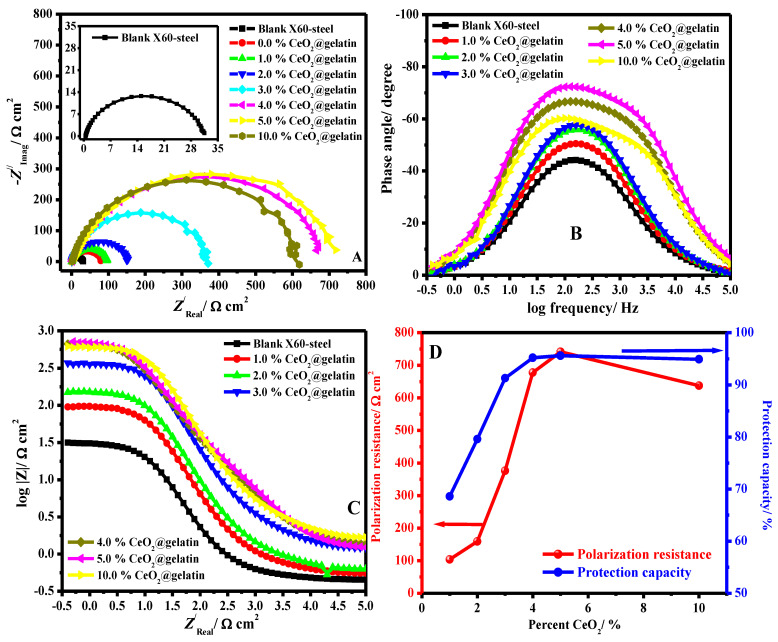
(**A**) Nyquist, (**B**) Bode, (**C**) Bode phase plots in 15% HCl solution for blank X60-steel and coated samples with different percent of % CeO_2_@gelatin at 50 °C and (**D**) Effect of CeO_2_ percent on the polarization resistance and protection capacity.

To explain the surface characteristics of the X60-steel and the kinetic processes of the metal, an impedance study was accompanied on the pristine and coated X60-steel in 15% HCl solution at 50 °C. The Nyquist (A), (B) Bode, (C) Bode phase plots in 15% HCl solution for blank X60–steel and coated samples with different percentages of % ceria@gelatin at 50 °C are depicted in Figure 7A–C respectively. The effect of CeO_2_ percent on the polarization resistance and protection capacity is presented in Figure 7D.

The Nyquist diagram (Figure 7A) is categorized by a single capacitive semicircle which resembles a unique time constant in the Bode profile (Figure 7B), indicating that the mechanism of corrosion is mostly organized by charge-transfer routes [5]. The deficient profile of the capacitive semicircle can be attributed to the coarseness and inhomogeneities of the substrate surface. The Nyquist diagram diameter in the occurrence of all coated films with various percent of ceria was greater than that of the pristine X60-steel surface. This suggests that the coating with % CeO_2_@gelatin produces a shielding layer on the metal surface, thus augmenting the steel surface impedance to electrochemical deterioration. This arc diameter upsurges with cumulative the ceria (CeO_2_) in the coating composition until 5% CeO_2_ then decrease to 10% CeO_2_ (Figure 7D), a suggestion that the protection of steel is in direct proportionality to the ceria dose. The % CeO_2_@gelatin coated films formed the defensive adsorbed layer on the steel interface by developing a strong bond among nitrogen atoms in the CeO_2_@gelatin film and the electrode interface. These films formed on the steel surface and efficiently block it from the aggressive solution [40]. The modulus of Bode impedance (Figure 7C) displays linear parts at middle frequencies. At intermediate frequencies, the linearity is more noticeable in the occurrence of the coated films, demonstrating higher slopes than the pristine steel [41].

**Figure 8:**
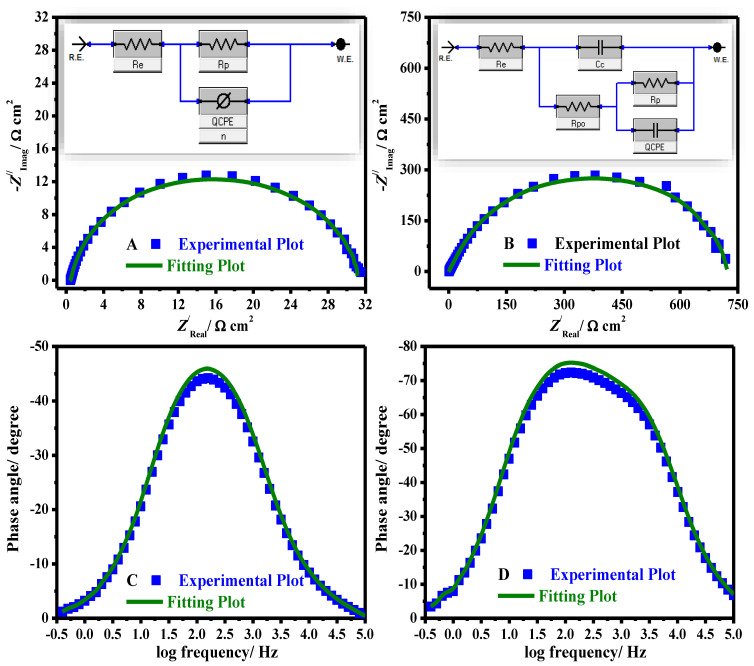
Comparison of experimental and fitting data (**A**,**B**) Nyquist, and (**C**,**D**) Bode phase plots in 15% HCl solution for uncoated (**A**,**C**) and coated using 5% CeO_2_@gelatin (**B**,**D**) systems. EEC for uncoated (inset **A**) and coated (inset **B**) systems.

A comparison of experimental and fitting data (A, B) Nyquist and (C, D) Bode phase plots in 15% HCl solution for uncoated (A, C) and coated (B, D) systems is presented in Figure 8.

The equivalent circuit (EEC) used to fit the impedance data is presented in Figure 8 (uncoated (inset A) and coated (inset B)). The fit precision ranged from 1.7 × 10^−5^ and 6.9 × 10^−5^ in all diagrams. The model used to fit the impedance data comprises *R_e_* (electrolyte resistance solution), *CPE* signifies constant phase-element, *R_p_* indicates the polarization resistance (*R_p_* = *R_ct_* (resistance of charge transfer) + *R_L_* (layer resistance)), i.e., a simple Randles EEC along with *R_po_* (resistance of electrical pores) and *C_c_* (pseudo-capacitance coating) in the occurrence of coated substrates. The computed electrochemical restrictions were fitted by Z-View software and are recorded in Table 2. The protection capacities (% *PC*) were estimated from Equation (6) [42]:

(6) $$PC/\%=\left(1-\frac{R_{p}^{0}}{R_{p}^{c}}\right)\times 100$$

where $R_{p}^{0}$ and $R_{p}^{c}$ characterize resistances of polarization in the case of pristine and coated systems, respectively.

Instead of performing as a pure capacitor, the double-layer designed by the film on the steel interface acts as *CPE*. To provide a more precise fitting, *CPE* was replaced for the capacitance element [43]. The *CPE* impedance is calculated from Equation: [44];

(7) $$Z_{CPE}=Y_{0}^{-1}{\left(j\omega \right)}^{-n}$$

where *Y*_0_ describes the *CPE* magnitude, *j* designates the $\sqrt{-1}$, *ω* represents angular frequency and *n* symbolizes the phase shift. The inferior value of *n* (Table 2) for pristine X60 –steel clarifies surface inhomogeneity designed from the metal coarsening surface owing to deterioration. However, in the case of % CeO_2_@gelatin coated films, the values of *n* were augmented, demonstrating lessening inhomogeneity of surface owing to the construction of combined coated films. Furthermore, the values of *R_po_* were found to be increased with CeO_2_ percent until 5% and then decreased. The maximum *R_po_* value was achieved for the specimen coated by 5% CeO_2_@gelatin film, confirming that this is the nominal permeable coating (Table 2). Commonly, the uniform and dense coatings act as an insulator with great resistances and small capacitances.

A substantial upsurge in the *R_p_* was detected in the case of the coated films (Table 2). The *R_p_*, upsurges with the increasing % CeO_2_ in gelatin matrix leading to the improved coating protection. This performance is owing to the construction of the surface layer on the steel interface by the coated films. The distinctive constructions of CeO_2_ nanoparticles impede the aggressive solution from entering the steel interface and upsurge the coating corrosion resistance, thus hindering more mass and charge transfer. In short, the *R_p_* and protection capacity order of different coating films during the whole examination route is as follows: 5% CeO_2_@gelatin (95.6%) > 4% CeO_2_@gelatin (95.2%) > 10% CeO_2_@gelatin (94.9%) > 3% CeO_2_@gelatin (91.3%) > 2% CeO_2_@gelatin (79.6%) > 1% CeO_2_@gelatin (68.6%).

The *CPE* values for the pristine and coated and the modified coatings are recorded in Table 2. In the case of coating films, the *CPE* values decrease as compared to the uncoated surface owing to the improved protection efficacy delivered by the CeO_2_@gelatin films. The lesser *CPE* value with a greater value of *R_p_* shows the larger corrosion impedance of the fabricated coating films [6]. The non-scratched coated specimens demonstrated at a well-clear capacitive response at significant frequencies, with higher values of the phase angle that supported very respectable barrier characteristics. It can be observed that the *Y*_0_ values declined significantly from 89.6 × 10^−7^ Ω^−1^ s^n^ cm^−2^ to 14.2 × 10^−7^, 11.3 × 10^−7^, 9.8 × 10^−7^, 7.1 × 10^−7^, 6.7 × 10^−7^, and 8.6 × 10^−7^ Ω^−1^ s^n^ cm^−2^ in the case of coated films with 1% CeO_2_@gelatin, 2% CeO_2_@gelatin, 3% CeO_2_@gelatin, 4% CeO_2_@gelatin, 5% CeO_2_@gelatin and 10% CeO_2_@gelatin, respectively. This decline in *CPE* findings is attributed to the upsurge in the electrical double layer thickness and/or the reduction in the local-dielectric constant, demonstrating that CeO_2_@gelatin functions by strong adsorption at the interface of the steel/medium [45]. The enhancement in *R_p_* is consistent with the upsurge in *R_po_* and the decrease in *CPE* values of the coating films.

### 3.5. Effect of Exposure Time (Stability of Coating Films)

**Figure 9:**
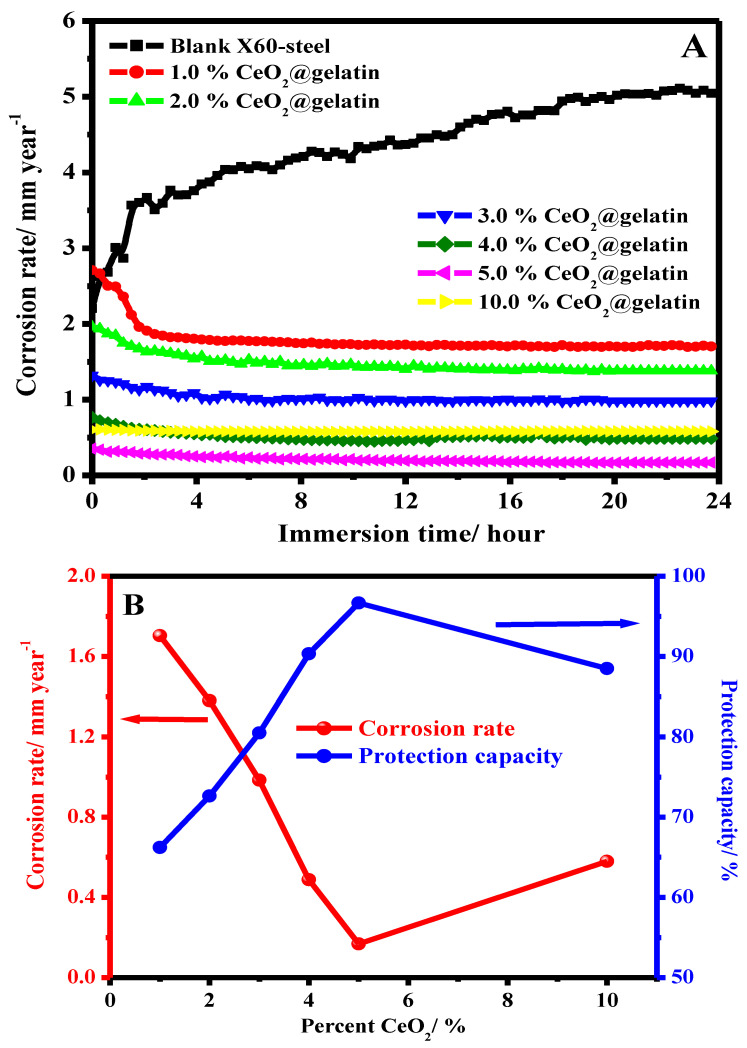
Effect of exposure time on the corrosion rate for blank X60-steel and coated samples with different percent of % CeO_2_@gelatin in 15% HCl solution at 50 °C (**A**,**B**) the effect of CeO_2_ percent on the corrosion rate and protection capacity after 24 h of exposure.

**Figure 10:**
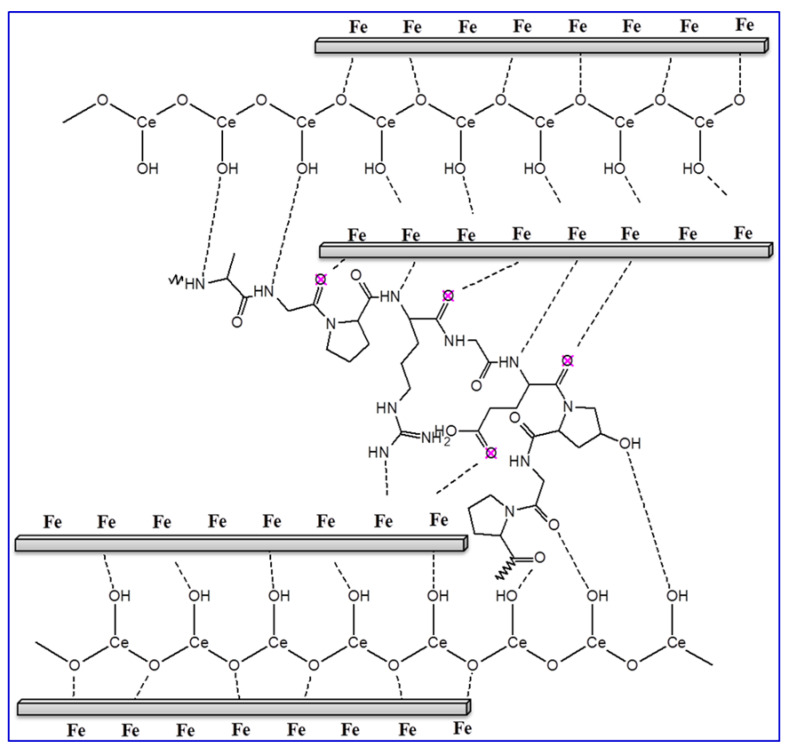
Schematic representation for the CeO_2_@gelatin molecule adsorption on the X60-steel substrate in HCl solution.

It is acknowledged that the protection proficiency and stability of coating films depend on their exposure time in the corrosive medium. In order to examine the influence of long-term dipping time on the protection capacity of coated X60-steel substrates in 15% HCl solution at 50 °C, the LPR corrosion rate is achieved. Figure 9A shows the effect of exposure time on the corrosion rate for blank X60-steel and coated samples with different percentages of % CeO_2_@gelatin in 15% HCl solution at 50 °C. For the uncoated X60-steel, it is apparent that (Figure 9A), the rate of corrosion (CR) gradually rises with exposure time; the CR began at 2.2056 to 5.0443 mm/year. The increase in CR could be owing to the galvanic impact between the Fe_3_C (iron carbide) and the ferrite phase (α-ferrite) [46]. Noticeably, the occurrence of coating layers on the X60-steel substrate significantly decreased the CR as the ceria percent increased up to 5% CeO_2_ and then increased. The CR dropped from 5.0443 to 0.152 mm/year in the case of samples coated with 5% CeO_2_@gelatin films after 24 h of exposure. It can be recognized as the robust adhesion of coating layers on the metal substrate. For coated films, throughout the dipping period of 24 h, the CR is still stable with exposure time as demonstrated in Figure 9A. This supports that % CeO_2_@gelatin films enhance the corrosion protection of the X60-steel surface. As could be observed from the Data in Figure 9B, as the exposure time augmented, the CR values declined and the protection capacity increased. It showed that the 5% CeO_2_@gelatin coated films have comparatively higher protection capacity with long-term anti-corrosive features. This is because, in the composite coating of 5% CeO_2_@gelatin, CeO_2_ nanoparticles are effectively compounded, giving full play to the anti-corrosion synergistic impact and efficiently protective X60-steel. The stability of coated films is related to the strong adsorption of gelatin molecules on the steel interface through the contribution of lone electron pairs of nitrogen and oxygen atoms to the vacant d-orbital of iron ions to produce a coated film of gelatin molecules functionalized by cerium oxide nanoparticles on the steel surface, as presented in Figure 10.

### 3.6. Surface Morphology

**Figure 11:**
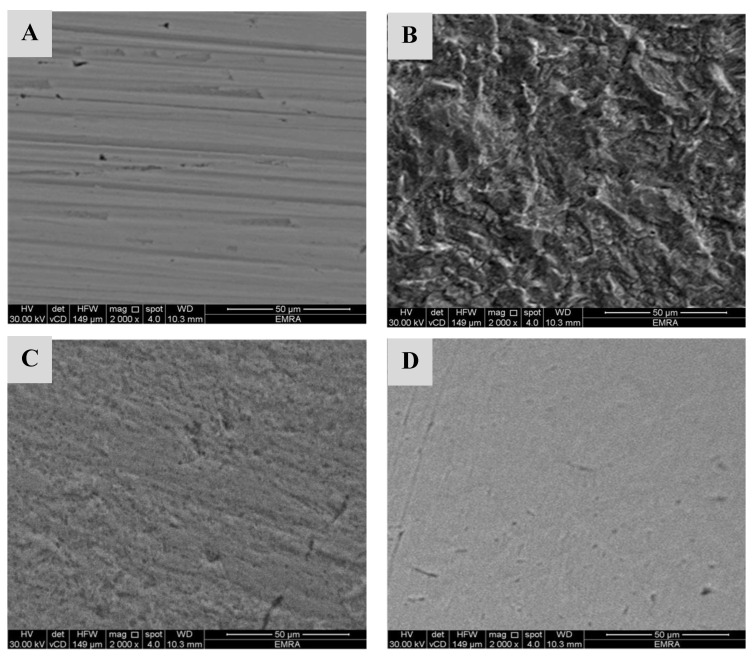
FESEM pictures of (**A**) pristine X60-steel, (**B**) uncoated X60-steel dipped in 15% HCl after 24 h, (**C**) X60-steel coated with 1% CeO_2_@gelatin dipped in 15% HCl after 24 h, and (**D**) X60-steel coated with 5% CeO_2_@gelatin dipped in 15% HCl after 24 h.

Scanning electron microscopy examinations were performed to confirm the protection capacity of CeO_2_@gelatin coated films on steel surfaces in the acidic medium. Figure 11A–D display the surface morphology (A) pristine X60-steel, (B) uncoated X60-steel dipped in 15% HCl after 24 h, (C) X60-steel coated with 1% CeO_2_@gelatin dipped in 15% HCl after 24 h, and (D) X60-steel coated with 5% CeO_2_@gelatin dipped in 15% HCl after 24 h. An SEM micrograph of the original steel electrode (uncoated) shows the brightness of the metal surface without any inclusions (Figure 11A). Figure 11B (uncoated steel) shows a rough surface with a great number of corrosions dispersed on the surface in a 15% HCl solution. The deterioration was significantly reduced in the case of coated % CeO_2_@gelatin films (Figure 11C,D), which illustrate a slicker and smoother surface. It has been described that a flatter surface topology is a result of protecting film construction by the fabricated coated films. In the case of a coated film with 5% CeO_2_@gelatin in the studied corrosive medium, more smooth, uniform, and brighter surface features can be observed (Figure 11D). This demonstrates a greater adhesion of the gelatin films in the occurrence of % CeO_2_ and further clarifies the higher protection capacity of gelatin films functionalized by 5% CeO_2_. Consequently, the coated % CeO_2_@gelatin films decrease the rate of corrosion of steel that takes place in the acidic chloride solution.

### 3.7. Computational Calculations (DFT)

**Figure 12:**
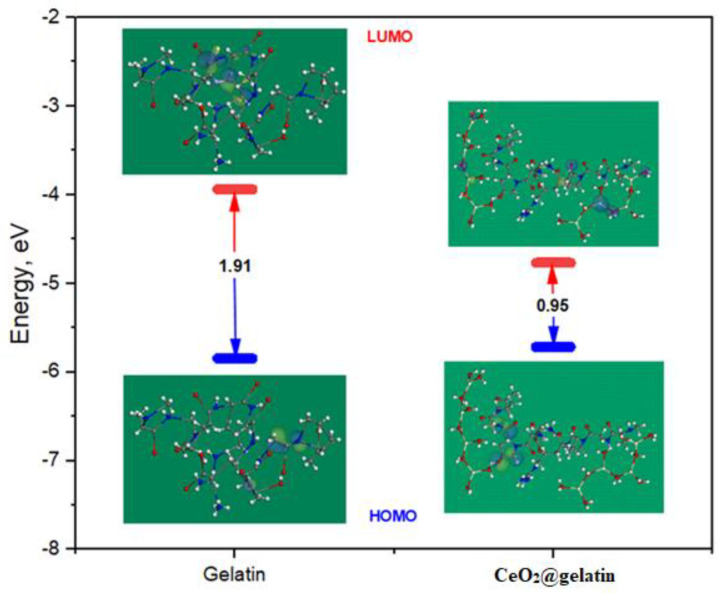
Energy diagram of the frontier molecular orbitals for the gelatin and CeO_2_@gelatin molecule using the DFT method.

Figure 12 represents the energy diagram of the frontier molecular orbitals (FMO) for the gelatin and CeO_2_@gelatin composite, and the correlated theoretical parameters are arranged in Table 3. As shown in Figure 12, it is observable that the *HOMO* level for the CeO_2_@gelatin molecule was placed on the nitrogen and oxygen atoms, which are favored sites for electrophilic attacks on the steel interface. In addition, the Δ*E* (energy gap) is a decisive parameter to reinforce the corrosion inhibition capacity of the inhibitor molecule which improves as the Δ*E* value is lessened [47]. As exhibited in Table 3, the CeO_2_@gelatin molecule has a smaller Δ*E* value (0.95 eV) than the CeO_2_ and gelatin molecules which enhances the inclination of the CeO_2_@gelatin molecule to be adsorbed on the steel interface.

Usually, the corrosion inhibitors have small values of electronegativity (*χ*) implying the inhibitor’s aptitude for electron provision to the metal surface [48], while the great values of electronegativity (*χ*) indicate the inhibitor’s ability to receive the electron from steel interface atoms (i.e., back-donation) and create a powerful bond with the steel surface [49]. As exhibited in Table 3, the electronegativity of CeO_2_, gelatin and CeO_2_@gelatin molecules are relatively high, revealing that the back-donation aptitude of the CeO_2_, gelatin and CeO_2_@gelatin molecules forms a strong bond with the steel surface.

Additionally, the softness (*σ*) and hardness (*η*) are important indicators to assess the stability and reactivity of the inhibitor molecule; soft molecules have protection capability greater than those of hard molecules through the effortless transfer of electrons to the steel interface forming the adsorbed layer, so it is considered as an efficient corrosion inhibitors [50]. As described in Table 3, the CeO_2_@gelatin molecule has a larger *σ* value (2.11) and a lower *η* value (0.47) than CeO_2_ and gelatin molecules describing efficient offering of electrons to the steel surface and excellent inhibition ability.

In addition, the fraction of electron transfer and Δ*E_back_*_-*donation*_ are pivotal directories to clarify the electron-donating or accepting ability of inhibitor molecules. Hence, if the Δ*N* values are >0, the electron transfer is feasible from inhibitor to steel interface atoms, whilst if the Δ*N* values are <0 zero, the electron transfer is possible from metal atoms to inhibitor molecules (i.e., back-donation) [51]. According to the Δ*N* value recorded in Table 3, the Δ*N* value for examined molecules is greater than zero (1.85) and higher than the CeO_2_ and gelatin molecules, demonstrating that the CeO_2_@gelatin molecule is able to contribute electrons to the steel surface. Furthermore, the Δ*E_back_*_-*donation*_ will be <0 when *η* > 0, the electron relocated to a molecule, followed by a back-donation from the molecule, and this is dynamically preferred [52]. In Table 3, the value of Δ***E****_back_*_-*donation*_ for CeO_2_, gelatin and CeO_2_@gelatin molecules is negative, which discloses that back-donation is desired for CeO_2_, gelatin, and CeO_2_@gelatin molecules molecule and constructs a hardy bond [48].

In addition, the dipole moment is a significant parameter that indicates the corrosion inhibition capacity of the inhibitor molecule [53]. Whereas the increase in dipole moment affords improved distortion energy and enhances the adsorption of the inhibitor molecule on the metal surface refers an strengthen in corrosion inhibition ability [54]. As shown in Table 3, the CeO_2_@gelatin molecule has a larger dipole moment value (13.54 Debye) than CeO_2_ and gelatin molecules which supports the likelihood of the CeO_2_@gelatin molecule to be adsorbed on the steel interface and the inhibition to be improved. Finally, the protection ability of the CeO_2_@gelatin molecule for the corrosion of the steel surface is associated with its molecular surface area. The corrosion prevention ability improved as the molecular area is augmented owing to the increase of the steel surface area covered with the inhibitor molecule [55]. As shown in Table 3, the CeO_2_@gelatin molecule had larger molecular surface area (3092.08 Å^2^) than the CeO_2_ and gelatin molecules, which reinforces the corrosion inhibition efficiency for the CeO_2_@gelatin molecule. The theoretical outcomes are in line with the experimental findings.

### 3.8. MC Simulations

**Figure 13:**
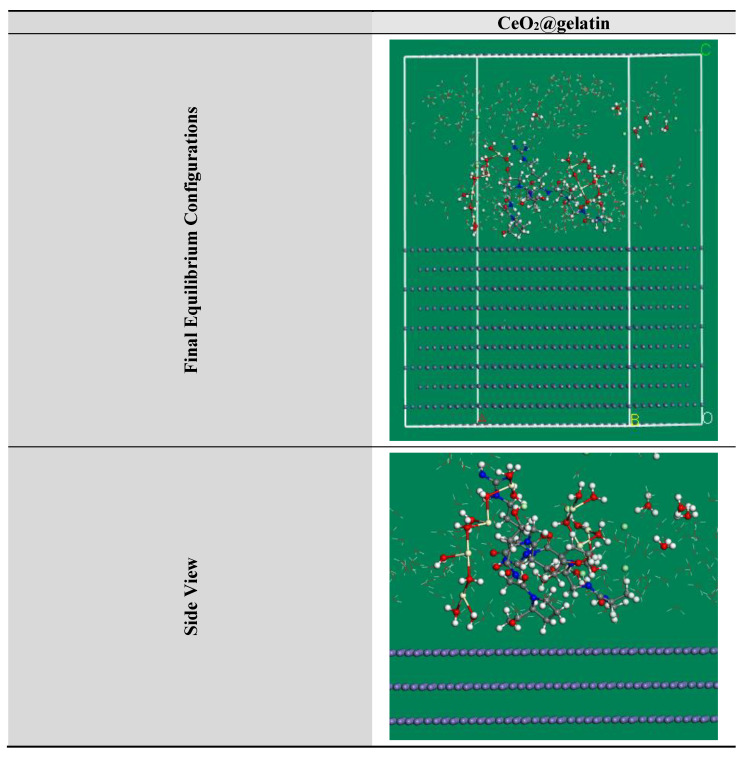

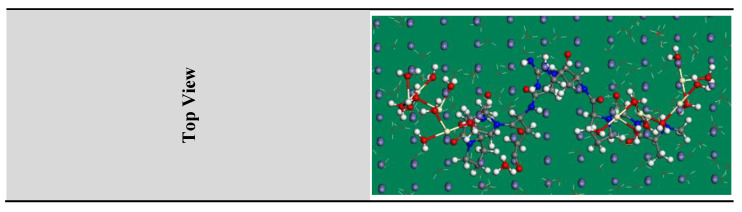
The highest proper adsorption arrangement for the CeO_2_@gelatin molecule on the Fe (1 1 0) substrate achieved by the adsorption locator module.

MC simulations were devoted to distinguishing the adsorption of the inhibitor molecule on the steel surface as well as indicating an obvious knowledge for the inhibition mechanism. Thus, Figure 13 shows the greatest suitable adsorption configurations for the CeO_2_@gelatin molecule on the steel interface in an acidic medium, which appeared in nearly flat disposition, proposing a development in the adsorption and maximum surface coverage [56]. Furthermore, the assessed outcomes for the adsorption energies from the MC simulations were shown in Table 4. Based on the outcomes in Table 4, the CeO_2_@gelatin molecule has a great negative adsorption energy value (−1813.21 kcal mol^−1^) which postulates the energetic adsorption of CeO_2_@gelatin molecule on the steel interface, generating a firm adsorbed film and protects the steel from the corrosion; these findings concur with the empirical findings [57]. In addition, Table 4 shows that the adsorption energy value for the CeO_2_@gelatin molecule for the pre-geometry optimization step (−1482.84 kcal mol^−1^) is high negative, and for post-geometry optimization step (−330.37 kcal mol^−1^) affirming a high protection ability for CeO_2_@gelatin molecule.

The d*E*_ads_/d*N*_i_ values elucidate the metal/adsorbates configuration energy if adsorbed species or the inhibitor molecule has been omitted [30]. The d*E_ads_*/d*N_i_* value for CeO_2_@gelatin molecule (−157.00 kcal mol^−1^) is higher negative than the d*E_ads_*/d*N_i_* values for water molecules, hydronium ions, and chloride ions are −15.49, −30.32, and −65.31 kcal mol^−1^, respectively. Consequently, the stronger adsorption of the CeO_2_@gelatin molecule than H_2_O molecules, hydronium ions, and chloride ions was detected, which improves the replacement of water molecules, hydronium ions, and chloride ions by the CeO_2_@gelatin molecule. Therefore, the CeO_2_@gelatin molecules are assertively adsorbed on the steel surface and construct an effective adsorbed defensive layer which protects the steel surface from corrosion, as declared by both experimental and theoretical studies.

## 4. Conclusions

Cerium oxide nanoparticles functionalized by gelatin were successfully designed with a different ceria percent. The composition and the structure of the as-prepared CeO_2_@gelatin nanocomposites were studied in terms of FE-SEM, EDX, TEM, chemical mapping, FT-IR, and (TGA) thermal analyses. The thermal characteristics of CeO_2_@gelatin approve the successful preparation of the CeO_2_@gelatin composite. The CeO_2_@gelatin nanocomposites were coated onto the X60 steel alloy surfaces, and their corrosion protection features were inspected by electrochemical (*E_ocp_*-time, EIS, PDP, and LPR corrosion rate) measurements in 15% HCl and compared with that of the pure gelatin films. The findings display that the prepared coating films offer good anti-corrosion protection to X60 steel alloy in the investigated acidizing oil well environments, with the protection degree depending on ceria percent in the content of coating films. Consequently, the 5% CeO_2_@gelatin sample had maximum *R*_p_ and lowermost *C_dl_*, signifying the best barrier property of this film with protection capacity (95.6%). FESEM examinations support the probability of protecting the X60 steel alloy against acidic corrosion by developing a defensive film of CeO_2_@gelatin using coatings. The outcomes of DFT calculation and MC simulation displayed that the coated films were strongly adsorbed onto the metal interface, and DFT parameters, including *E_HOMO_*, *E_LUMO_*, and Δ*E*, were in agreement with the empirical findings, which assistance clarify the adsorption mechanism of the CeO_2_@gelatin on the iron surface.

## Figures and Tables

**Table 1:** Tafel parameters for blank and coated X60-steel in 15% HCl solution at 50 °C.

Samples Description	*E_cor_*/ mV vs. (Ag/AgCl)	*j_cor_*/ A cm^−2^ × 10^−6^ ±SD	*β_a_*/ mV dec^−1^	*-β_c_*/ mV dec^−1^	*η_Pc_*/%
Blank X60-steel	−418 ± 3	398.1 ± 24.7	195.4	382.5	-
Pure gelatin (0.0% CeO_2_)	−416	159.2 ± 21.1	221.2	401.3	60.0
1.0% CeO_2_@gelatin	−407 ± 5	118.23 ± 11.3	224.9	426.8	70.3
2.0% CeO_2_@gelatin	−412 ± 4	73.25 ± 5.8	215.5	431.2	81.6
3.0% CeO_2_@gelatin	−404 ± 6	25.47 ± 1.5	220.1	396.5	93.6
4.0% CeO_2_@gelatin	−389 ± 4	9.95 ± 1.1	210.2	414.6	97.5
5.0% CeO_2_@gelatin	−385 ± 9	7.16 ± 0.71	224.3	433.9	98.2
10.0% CeO_2_@gelatin	−378 ± 8	14.33 ± 1.2	208.8	423.4	96.4

**Table 2:** EIS fitting parameters for blank and coated X60-steel in 15% HCl solution at 50 °C.

Samples Description	*R_e_*/ Ω cm^2^	*CPE_coat_ (Q_coat_)*		R*po/* Ω cm^2^ ±SD	*Rp/*Ω cm^2^ ±SD	*CPEdl (Qdl)*		*θ*	*η_PC_/%*
		*Y*_0_/ Ω^−1^ s^n^ cm^−2^	*n*			*Y*_0_/ Ω^−1^ s^n^ cm^−2^	*n*		
Blank X60-steel	0.43	--	---	---	32.5 ± 2.1	89.6 × 10^−7^	0.725	--	---
Pure gelatin (0.0% CeO_2_)	0.65	81.6 × 10^−7^	0.823	14.4 ± 1.2	80.2 ± 4.7	18.4 × 10^−7^	0.856	0.594	59.4
1.0% CeO_2_@gelatin	0.71	67.2 × 10^−7^	0.834	17.4 ± 1.3	103.8 ± 6.6	14.2 × 10^−7^	0.849	0.686	68.6
2.0% CeO_2_@gelatin	1.10	41.7 × 10^−7^	0.852	21.2 ± 1.5	159.4 ± 11.3	11.3 × 10^−7^	0.831	0.796	79.6
3.0% CeO_2_@gelatin	1.35	32.9 × 10^−7^	0.843	29.3 ± 2.3	376.1 ± 16.7	9.8 × 10^−7^	0.879	0.913	91.3
4.0% CeO_2_@gelatin	1.92	21.4 × 10^−7^	0.815	42.2 ± 3.5	677.9 ± 29.8	7.1 × 10^−7^	0.895	0.952	95.2
5.0% CeO_2_@gelatin	2.43	19.7 × 10^−7^	0.834	49.4 ± 3.8	741.3 ± 43.2	6.7 × 10^−7^	0.894	0.956	95.6
10.0% CeO_2_@gelatin	2.88	23.8 × 10^−7^	0.861	44.5 ± 3.1	637.7 ± 33.1	8.6 × 10^−7^	0.873	0.949	94.9

**Table 3:** DFT parameters for CeO_2_, gelatin and CeO_2_@gelatin molecules.

Parameters	CeO_2_	Gelatin	CeO_2_@gelatin
*E_HOMO_* (eV)	−8.48	−5.85	−5.72
*E_LUMO_* (eV)	−1.71	−3.94	−4.77
Δ*E* = *E_LUMO_* − *E_HOMO_* (eV)	6.77	1.91	0.95
Electronegativity (*χ*)	5.10	4.90	5.25
Global hardness (*η*)	3.39	0.95	0.47
Global softness (*σ*)	0.30	1.05	2.11
The number of electrons transferred (Δ*N*)	0.28	1.10	1.85
∆*E_back-donation_*	−0.85	−0.24	−0.12
Dipole moments (*µ*) Debye	2.99	5.48	13.54
Molecular surface area (Å^2^)	427.35	2157.28	3092.08

**Table 4:** Data and descriptors computed by the MC simulations for the adsorption of the CeO_2_@gelatin molecule on Fe (1 1 0).

Corrosion Systems	Adsorption Energy/ kcal mol^−1^	Rigid Adsorption Energy/ kcal mol^−1^	Deformation Energy/ kcal mol^−1^	d*E_ads_*/dN*_i_*: Inhibitor kcal mol^−1^	d*E_ads_*/d*N_i_*: Cl^−^ ions kcal mol^−1^	d*E_ads_*/d*N_i_*: Hydronium kcal mol^−1^	d*E_ads_*/d*N_i_*: Water kcal mol^−1^
Fe (110)	−1813.21	−1482.84	−330.37	−157.00	−65.31	−30.32	−15.49
CeO_2_@gelatin							
Water							
Hydronium							
Cl^−^ ions							

## Data Availability

The raw/processed data generated in this work are available upon request from the corresponding.
